# Evolution of insulin sensitivity and its variability in out-of-hospital cardiac arrest (OHCA) patients treated with hypothermia

**DOI:** 10.1186/s13054-014-0586-x

**Published:** 2014-10-28

**Authors:** Azurahisham Sah Pri, J Geoffrey Chase, Christopher G Pretty, Geoffrey M Shaw, Jean-Charles Preiser, Jean-Louis Vincent, Mauro Oddo, Fabio S Taccone, Sophie Penning, Thomas Desaive

**Affiliations:** Centre for Bio-Engineering, Department of Mechanical Engineering, University of Canterbury, 20 Kirkwood Avenue, Christchurch, 8140 New Zealand; Department of Intensive Care, Christchurch Hospital, Riccarton Avenue, Christchurch, 8140 New Zealand; Department of Intensive Care, Erasme University Hospital (CUB), University of Brussels, Route de Lennik 808, 1070 Brussels, Belgium; Department of Intensive Care, Lausanne University Hospital (CHUV), Rue du Bugnon 46, 1011 Lausanne, Switzerland; Cardiovascular Research Center, Universite de Liege, Allée du 6 Août 17, B4000 Liege, Belgium

## Abstract

**Introduction:**

Therapeutic hypothermia (TH) is often used to treat out-of-hospital cardiac arrest (OHCA) patients who also often simultaneously receive insulin for stress-induced hyperglycaemia. However, the impact of TH on systemic metabolism and insulin resistance in critical illness is unknown. This study analyses the impact of TH on metabolism, including the evolution of insulin sensitivity (S_I_) and its variability, in patients with coma after OHCA.

**Methods:**

This study uses a clinically validated, model-based measure of S_I_. Insulin sensitivity was identified hourly using retrospective data from 200 post-cardiac arrest patients (8,522 hours) treated with TH, shortly after admission to the intensive care unit (ICU). Blood glucose and body temperature readings were taken every one to two hours. Data were divided into three periods: 1) cool (T <35°C); 2) an idle period of two hours as normothermia was re-established; and 3) warm (T >37°C). A maximum of 24 hours each for the cool and warm periods was considered. The impact of each condition on S_I_ is analysed per cohort and per patient for both level and hour-to-hour variability, between periods and in six-hour blocks.

**Results:**

Cohort and per-patient median S_I_ levels increase consistently by 35% to 70% and 26% to 59% (*P* <0.001) respectively from cool to warm. Conversely, cohort and per-patient S_I_ variability decreased by 11.1% to 33.6% (*P* <0.001) for the first 12 hours of treatment. However, S_I_ variability increases between the 18th and 30th hours over the cool to warm transition, before continuing to decrease afterward.

**Conclusions:**

OCHA patients treated with TH have significantly lower and more variable S_I_ during the cool period, compared to the later warm period. As treatment continues, S_I_ level rises, and variability decreases consistently except for a large, significant increase during the cool to warm transition. These results demonstrate increased resistance to insulin during mild induced hypothermia. Our study might have important implications for glycaemic control during targeted temperature management.

## Introduction

Hyperglycaemia is prevalent in critical care [1–4] and increases the risks of further complications and mortality [1,4,5]. Glycaemic control has shown benefits in reducing mortality and morbidity [4,6,7]. However, many studies have found it difficult to reproduce these results [8–10] due in part to metabolic variability [11]. Out-of-hospital cardiac arrest (OHCA) patients often experience hyperglycaemia [12,13]. These patients belong to one group who can be highly insulin resistant and variable, particularly on the first two days of stay [14], as well as those who may particularly benefit from glycaemic control [4].

Therapeutic hypothermia (TH) is often used with OHCA patients to protect against brain injury [15,16], which leads to a lowering of metabolic rate, reduces plasma insulin, induces insulin resistance and alters blood glucose homeostasis [17]. One of the adverse events associated with hypothermic therapy is a decrease in insulin sensitivity and endogenous insulin secretion [18]. However, this decrease may not be observable in a cohort who is already highly insulin resistant and variable [14]. Hence, understanding metabolic evolution and variability would enable safer and more accurate glycaemic control using insulin in this cohort. This study analyses the evolution of a clinically validated model-based measure of insulin sensitivity (S_I_) in OHCA patients to assess the impact of hypothermia therapy.

## Methods

### Patients and data

A retrospective analysis of glycaemic control data from 200 OHCA patients (8,522 hours) treated with TH, shortly after admission to intensive care. Data was obtained from intensive care units (ICUs) at Christchurch Hospital, New Zealand, at Erasme Hospital, Belgium, and CHUV-Lausanne Hospital, Switzerland. Patients from Christchurch Hospital (N = 20) were on the specialized relative insulin and nutrition titration (SPRINT) glycaemic control protocol [7], whereas the remaining 180 patients from Erasme (N = 99) and Lausanne (N = 81) hospitals were on local glycaemic control protocols and included in an institutional database (2008 to 2012).

Blood glucose (BG) and temperature readings were taken one to two hourly. Data were divided into three periods: 1) cool (T <35°C); 2) an idle period of two hours as normothermia was restored; and 3) warm (T >37°C). A maximum of 24 contiguous hours and a minimum of 15 hours for each period were considered, ensuring a balance of contiguous data between periods. Overall demographics are shown in Table 1.

**Table 1:** **Demographic data and treatment information for both the cool and warm periods**

**Variables**	**Value**	
	**Cool**	**Warm**
Total patients, number (n)	200	
Median age, years	61 [51, 72]	
Female gender, number (%)	40 (20.6%)	
ICU mortality, number (%)	85 (45.6%)	
Diabetes status, number (%)	26 (13.0%)	
Total treatment, hours (h)	4219	4303
Blood glucose, median (mmol/L)	7.6 [6.3,9.7]	6.8 [5.9,8.0]
Insulin rate, median (U/hr)	3.4 [1.3,8.0]	3.5 [1.6,7.0]
Glucose rate, median (g/hr)	2.7 [1.0,5.3]	5.4 [2.7,8.1]

IQR: [interquartile range].

Glycaemic targets while treating OHCA patients in the three units were very similar and overlapped. The SPRINT protocol, used in the Christchurch Hospital ICU, targeted 4.0 to 7.0 mmol/L [7]. The protocol used in both the Erasme and Lausanne ICUs differed from SPRINT, but targeted 6.0 to 8.0 mmol/L [17]. Although two different protocols were used in the three units, the targets were very similar and within the relatively tight 4.0 to 8.0 mmol/L range.

Audit of the clinical data from SPRINT was given by the Upper South B Regional Ethics Committee and for the data study by Taccone *et al*. [17]. No approval was required as it was also a retrospective audit.

### Model-based insulin sensitivity

Model-based S_I_ in this study is a patient-specific parameter describing the overall whole-body effect of insulin. S_I_ is identified for each hour, for each patient using a clinically validated glucose-insulin model [19–23]. The key model equations are defined:

1 $$ \dot{G}=-{p}_G.G(t)-{S}_I(t).G(t).\frac{Q(t)}{1+{\alpha}_GQ(t)}+\frac{P(t)+EGP-CNS}{V_G} $$

2 $$ \dot{I}=-{n}_KI(t)-\frac{n_LI(t)}{1+{\alpha}_II(t)}-{n}_I\left(I(t)-Q(t)\right)+\frac{u_{ex}(t)}{V_I}+\left(1-{X}_L\right)\frac{u_{en}(t)}{V_I} $$

3 $$ \dot{Q}={n}_I\left(I(t)-Q(t)\right)-{n}_C\frac{Q(t)}{1+{\alpha}_GQ(t)} $$

Where *G(t)* represents the concentration of blood glucose (mmol/L). *I(t)* and *Q(t)* represent the plasma insulin and insulin interstitial concentrations (mU/L) respectively. Model parameters, rates and constants in this model were as fully defined in [21,24].

Model-based S_I_ is identified hourly from patient data, producing an hourly piece-wise constant profile [25], capturing the whole-body glycaemic response to exogenous insulin and nutrition. The validity and independence of this patient-specific parameter have been validated using data from independent, clinically matched cohorts [19], in comparison to gold-standard insulin sensitivity tests [22] and in clinical glycaemic control [20,23].

### Analyses and metrics

S_I_ level and variability during the cool (T ≤35°C) and warm (T >35°C) periods are analysed on per-cohort and per-patient bases using six-hour blocks of data as per Table 2. S_I_ level is compared between blocks as a cohort median and by per-patient median S_I_. Similarly, S_I_ variability is calculated as the hour-to-hour percentage change in S_I_ (∆%S_I_) and is analysed per cohort for each block.

**Table 2:** **Descriptions of six-hour blocks for data analysis**

**Day**	**Period**	**Analysis**	**Block**	**Hour range**
1	Cool	6-hour block	1	0 – 6 hours
			2	6 – 12 hours
			3	12 – 18 hours
			4	18 – 24 hours
Idle 2-hour period in between cool and warm				
2	Warm	6-hour block	5	24 – 30 hours
			6	30 – 36 hours
			7	36 – 42 hours
			8	42 – 48 hours

4 $$ \%\Delta \mathrm{S}\mathrm{I}=\frac{\left({S}_{I_{n+1}}-{S}_{I_n}\right)}{S_{I_n}} \times 100 $$

The use of percentage change, rather than absolute change, normalises the metric so patients with differing S_I_ levels can be compared fairly.

Bagshaw *et al*. [26] reported an association between both hypoglycaemia and BG variability with mortality during the first 24 hours of ICU stay. Thus, the acute evolution of S_I_ over the first day using six-hour blocks was analysed as S_I_ variability is a key contributor to BG variability. For the cohort analysis, S_I_ and ∆%S_I_ data from all patients was grouped into each appropriate time block. Median values for each time block were calculated for comparison to the previous block, thus capturing overall cohort changes over time in level and hour-to-hour variability.

For the per-patient analysis, the median value of S_I_ and the interquartile range (IQR) of ∆% S_I_ were calculated for each patient, for each time block. The IQR captures the width or degree of variability for a given patient within each six-hour block. Thus, a reduction in the IQR of ∆% S_I_ over time would indicate a reduction in hour-to-hour variability for a given patient.

S_I_ level and variability are non-Gaussian and thus compared using non-parametric statistics and cumulative distribution functions (CDFs). CDFs are particularly useful as they show the entire distribution that is often summarised as a median and IQR. The CDF for a given value of the independent variable (for example S_I_ = x) describes the probability of observing a value less than or equal to x. All distributed data were compared using a Wilcoxon rank-sum test (Mann–Whitney *U* test), except for S_I_ variability results. S_I_ variability was compared using the Kolmogorov-Smirnov (KS) test as it has greater power to detect differences in the shape of distributions when median values are similar. In all cases, *P* <0.05 is considered statistically significant.

## Results

### S_I_ level analyses

**Figure 1:**
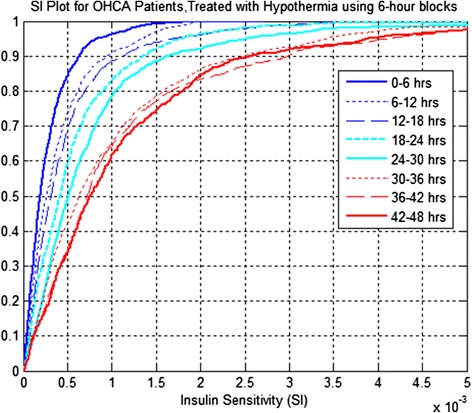
**Insulin sensitivity (S**_**I**_ **) level distribution per cohort for out-of-hospital cardiac arrest (OHCA) patients, treated with therapeutic hypothermia (TH) using six-hour blocks for both cool and warm periods.**

**Figure 2:**
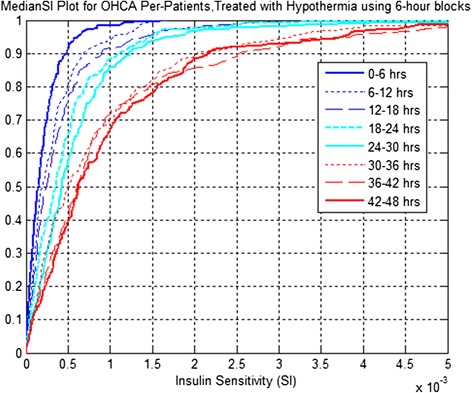
**Insulin sensitivity (S**_**I**_ **) level distribution per patient for out-of-hospital cardiac arrest (OHCA) patients, treated with therapeutic hypothermia (TH) using six-hour blocks for both cool and warm periods.**

Figures 1 and 2 present the CDFs of hourly S_I_ level by cohort and median S_I_ per patient, respectively, using six-hour blocks. Table 3 presents the increase in median insulin sensitivity and corresponding *P* values between successive time blocks.

**Table 3:** **Increasing cohort and per-patient median S**_**I**_ **during cool and warm periods as per six-hour blocks of data, where the** ***P*** **values compare successive six-hour blocks as shown in the first column for both the overall cohort and per-patient median values**

**S**_**I**_ **level**	**Cohort analysis**		**Per-patient analysis**	
**analysis**	**% S**_**I**_ **median increase**	***P*** **value**	**% S**_**I**_ **median increase**	***P*** **value**
**(6-hr blocks)**				
Block 1–2 (C)	35.1	<0.05	26.4	<0.05
**(0–6 vs. 6–12 hr)**				
Block 2–3 (C)	19.2	<0.05	31.1	<0.05
**(6–12 vs. 12–18 hr)**				
Block 3–4 (C)	31.8	<0.05	42.4	<0.05
**(12–18 vs. 18–24 hr)**				
Block 4–5 (C-W)	23.4	<0.05	18.3	<0.05
**(18–24 vs. 24–30 hr)**				
Block 5–6 (W)	23.9	<0.05	23.2	<0.05
**(24–30 vs. 30–36 hr)**				
Block 6–7 (W)	13.1	0.06	15.8	0.2
**(30–36 vs. 36–42 hr)**				
Block 7–8 (W)	4.4	0.4	3.2	0.5
**(36–42 vs. 42–48 hr)**				

*P* values are calculated using Wilcoxon rank-sum test. S_I_, insulin sensitivity metric (model-based).

The results suggest that S_I_ increases for the cohort and per patient are statistically significant for the first 36 hours (*P* <0.05) in both cases.

**Figure 3:**
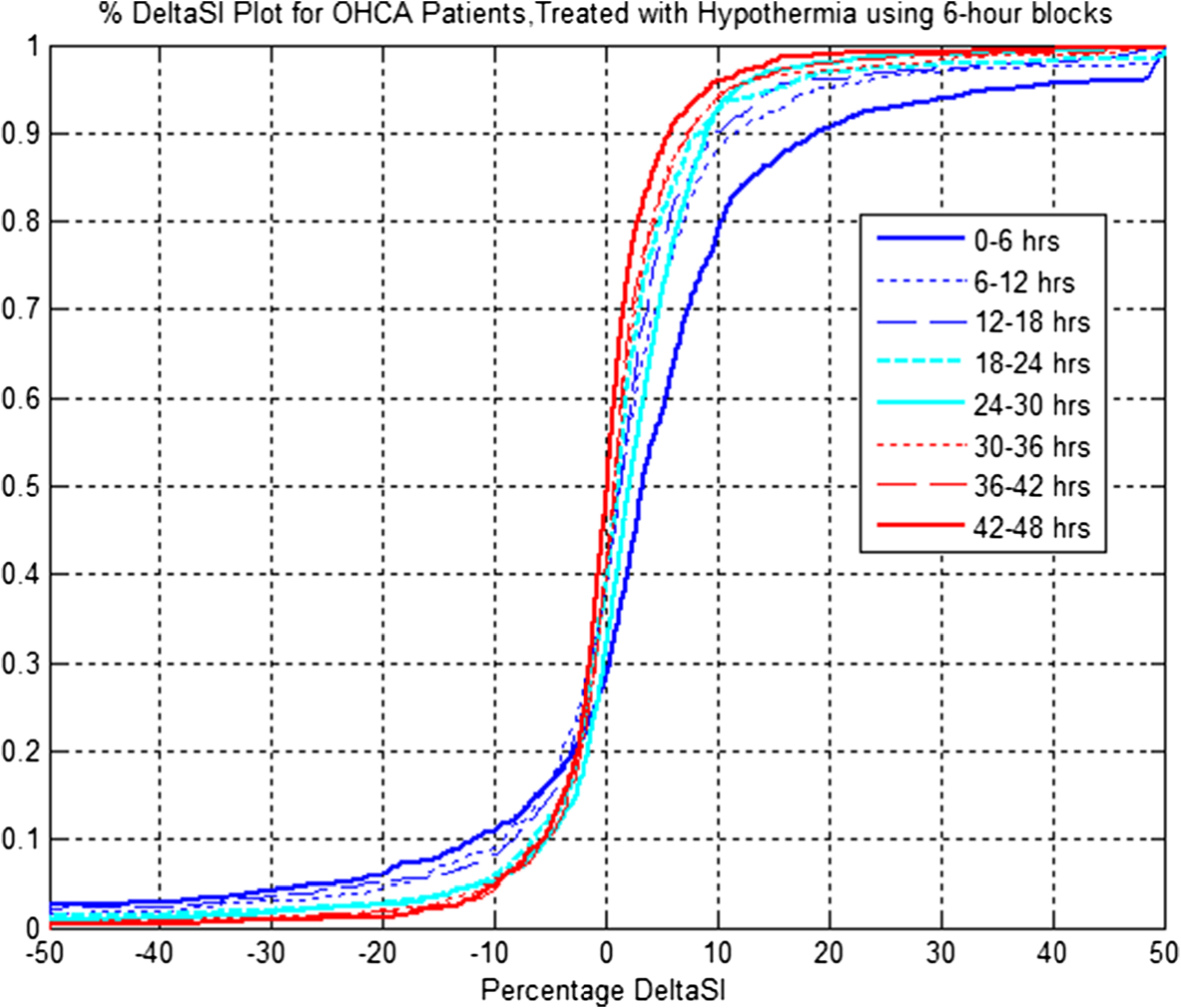
**Insulin sensitivity variability distribution (%∆S**_**I**_ **) per cohort for out-of-hospital cardiac arrest (OHCA) patients, treated with therapeutic hypothermia (TH) using six-hour blocks for both cool and warm periods.**

Results in Figure 2, Figure 3 and Table 3 are further reflected in Table 4, which shows that S_I_ increases for a large proportion of patients between the six-hour blocks over the first 36 hours of ICU stay. Table 4 also shows that after 48 hours of treatment, only 86% of patients show rise in S_I_ from the first six hours. Thus, while the general trend is obvious for increasing S_I,_ it is not guaranteed for all patients. Equally, these increases decelerate in terms of number of patients with increasing S_I_ over time, going from left to right in the table.

**Table 4:** **Proportion of patients for whom median insulin sensitivity increases between the blocks indicated in the row and columns**

	**6 -12 hr**	**12-18 hr**	**18-24 hr**	**24-30 hr**	**30-36 hr**	**36-42 hr**	**42-48 hr**
0 – 6 hr	0.72	0.74	0.79	0.83	0.84	0.85	0.86
6 – 12 hr		0.66	0.72	0.74	0.76	0.82	0.82
12 – 18 hr			0.69	0.70	0.75	0.79	0.79
18 – 24 hr				0.66	0.65	0.70	0.72
24 – 30 hr					0.64	0.68	0.66
30 – 36 hr						0.58	0.61
36 – 42 hr							0.52

### S_I_ variability analyses

**Figure 4:**
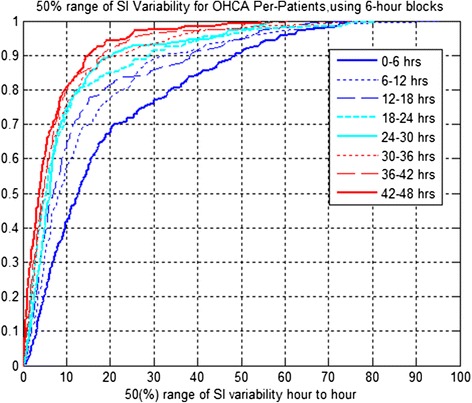
**Per-patient 50% range of S**_**I**_ **variability distribution of out-of-hospital cardiac arrest (OHCA) patients, treated with therapeutic hypothermia (TH) using six-hour blocks for both cool and warm periods.**

Figures 3 and 4 present the CDFs for changes in S_I_ (%∆S_I_) for six-hourly blocks per cohort and 50% range of S_I_ variability per patient, respectively. Table 5 presents the reductions between successive blocks.

**Table 5:** **Reductions in the interquartile range and median S**_**I**_ **per patient range of hour-to-hour percentage S**_**I**_ **change over time during cool and warm periods as per six-hour blocks of data, where the** ***P*** **values compare successive six-hour blocks as shown in the first column for both the overall cohort and per-patient median values**

**S**_**I**_ **variability**	**Cohort analysis**		**Per-patient analysis**	
**analysis**	**% reduction of IQR**	***P*** **value**	**% median decrease**	***P*** **value**
**[6-hr blocks]**				
Block 1–2 (C)	11.1	<0.05	33.6	<0.05
**(0–6 vs. 6–12 hr)**				
Block 2–3 (C)	20.7	<0.05	15.8	<0.05
**(6–12 vs. 12–18 hr)**				
Block 3–4 (C)	14.4	<0.05	22.6	<0.05
**(12–18 vs. 18–24 hr)**				
Block 4–5 (C-W)	−19.7	<0.05	−14.9	<0.05
**(18–24 vs. 24–30 hr)**				
Block 5–6 (W)	23.1	<0.05	26.4	0.05
**(24–30 vs. 30–36 hr)**				
Block 6–7 (W)	4.6	<0.05	0.8	0.05
**(30–36 vs. 36–42 hr)**				
Block 7–8 (W)	13.0	0.08	17.1	0.06
**(36–42 vs. 42–48 hr)**				

*P* values are calculated using the Kolmogorov-Smirnov test. S_I_, insulin sensitivity metric (model-based); IQR, interquartile range.

Cohort and per-patient variability decreases for the first 24 hours. However, it increases across the cool to warm transition, indicating some potential stress across the cool to warm transition with negative reductions. The decreasing trend returns for all subsequent blocks. The results suggest that %∆S_I_ decreases per cohort and per patient are statistically significant (*P* <0.05) for the first 36 hours in both cases.

## Discussion

### Insulin sensitivity level

The S_I_ level results for both per-cohort and per-patient analysis suggest that OHCA patients undergoing TH treatment have significantly lower S_I_ during the earlier cool period on day 1 than the later warm period on day 2. Both results determine the general trend for overall increasing S_I_ level for critically ill patients over time and are consistent with other ICU studies [14,27]. Further analysis shows that the increase in S_I_ level during the first 36 hours are large and statistically significant for this cohort. The rapid increases in S_I_ level for the first 36 hours is likely due to significant restart of human physiological systems and metabolic activities for these patients [13]. After 36 hours, the rapid S_I_ increase abates as the patients’ metabolism improves and becomes more stable.

### Insulin sensitivity variability

Both per-cohort and per-patient analysis suggest that OHCA patients undergoing TH treatment have high initial variability that decreases over the first 36 hours. However, the cool to warm transition at 24 hours shows an increase in variability likely due to the change of physiological conditions as body temperature increases from cool to warm between 18 and 36 hours. The lower decrease in S_I_ variability after the 36th hour onward suggests that the patients’ metabolic condition has improved and become more stable.

Further analysis and comparison of S_I_ variability between general ICU patients [14] and OHCA patients treated with TH shows that the main difference between them is the S_I_ variability increase during the cool to warm transition period for the latter cohort. These S_I_ variability results do not follow the same trend with other general ICU studies by Pretty *et al*. [14], and it is a unique finding for this cohort that could significantly impact glycaemic control and safety from hypoglycaemia.

### Implications for glycaemic control

Clinically, these results have significant implications for managing glycaemia. Increased S_I_ variability leads to increased variability in BG level for a given insulin intervention [11]. With low and variable insulin sensitivity, glycaemic levels might appear to remain unchanged and difficult to control effectively with exogenous insulin. This situation may result in increased glycaemic variability as well as an increased risk of hyperglycaemia and hypoglycaemia during the first 36 hours of treatment due to greater hour-to-hour S_I_ variability with increased insulin resistance [17]. Thus, since glycaemic variability and hypoglycaemia are independent risk factors for the critically ill, it is important to understand and manage these patient-specific dynamics, especially those unique to a cohort, when implementing glycaemic control. This outcome is particularly important when OHCA patients transition from cool to warm. These results may also generalise to other areas where glycaemic control is applied to hypothermic patients, such as in the operating theatre.

There are several ways that this low and variable insulin sensitivity could be managed during glycaemic control. Reducing exogenous insulin doses, coupled with modulation of the glucose content of nutrition would diminish the impact of sudden changes of insulin sensitivity on glycaemic outcome. Equally, increased BG measurement frequency could improve control and reduce glycaemic variability. Accepting higher glycaemic targets during periods of increased variability would trade off a reduced risk of hypoglycaemia against increased hyperglycaemia. Ultimately, the preferred method for any unit may be influenced by practical considerations, such as clinical workload.

### Limitations

The parameters used in the glucose insulin system model are based on general ICU patients with normal body temperature conditions. Thus, the insulin sensitivity values derived during the cool period could be biased by modelling errors or unmodelled effects. However, as noted previously, the validity and independence of this patient-specific parameter has been validated using data from clinically matched cohorts and has been shown to correlate well in gold-standard insulin sensitivity tests.

Insulin sensitivity variability is a key contributor to glycaemic variability. Sechterberger *et al*. [28] showed an association between high glycaemic variability and mortality is not present in diabetic cohorts. Thus, a subgroup analysis of diabetic OHCA patients in this study would be very interesting. However, only 26 of 200 (13%) OHCA patients in this study had previously diagnosed diabetes (Table 1), which is too few to enable a reliable analysis with these methods. Additionally, in this particular cohort, undiagnosed diabetes or impaired glucose regulation [29] may confound such a subgroup.

## Conclusions

This study analyses the metabolic evolution of OHCA patients treated with TH. These analyses characterise the metabolic impact of TH treatment on the level and variability of insulin sensitivity to inform control.

Two main conclusions are drawn as a result for these cohorts.

i) S_I_ level is much lower during TH and consistently increases over time, during both cool and warm periods.
ii) Insulin sensitivity is more variable during the cool period and shows contrasting behaviour during the cool to warm transition period between 18 and 30 hours, which indicates that there are major changes in physiology and metabolic conditions between cool and warm as influenced by human body temperature. Otherwise, it decreases over time.

Finally, this study shows the need for patient-specific glycaemic management to ensure good control and safety during treatment. These results have significant potential clinical impact on the metabolic treatment of these patients, and changes in clinical therapy are required to safely treat patients as they transition from cool to warm.

## Key messages

- OCHA patients treated with TH have significantly lower and highly variable S_I_ during the first 24 hours of the cool period, compared to the later warm period in their ICU stay.
- There is an overall trend of increasing S_I_ over the first 36 hours, both per-cohort and per-patient results.
- S_I_ variability decreases consistently over time, except for a large, statistically significant increase during the cool to warm transition at 24 hours.
- This increase requires special consideration for glycaemic control as it increases risk of hypoglycaemia, BG variability and thus mortality.

## Abbreviations

%∆SI: hour-to-hour percentage changes in insulin sensitivity
BG: blood glucose
CDF: cumulative distribution function
ICU: intensive care unit
IQR: interquartile range
KS: Kolmogorov-Smirnov (test)
OHCA: out-of-hospital cardiac arrest
S_I_: insulin sensitivity metric (model-based)
SPRINT: specialized relative insulin and nutrition titration
TH: therapeutic hypothermia
